# Magnitude and risk of neonatal death in neonatal intensive care unit at referral hospital in Godeo Zone: a prospective cohort study

**DOI:** 10.11604/pamj.2021.38.201.20650

**Published:** 2021-02-22

**Authors:** Akine Eshete, Henok Tadesse, Tizalegn Tesfaye, Silesh Abiy

**Affiliations:** 1 College of Health Sciences and Medicine, Department of Public Health, Debre Birhan University, Debre Birhan, Ethiopia,; 2 College of Health Sciences and Medicine, Department of Public Health, Dilla University, Dilla, Ethiopia,; 3 College of Health Sciences and Medicine, Department of Anesthesiology, Dilla University, Dilla, Ethiopia

**Keywords:** Magnitude, neonatal death, risk factors, neonatal intensive care unit, Gedeo Zone-Ethiopia

## Abstract

**Introduction:**

even though newborn health is apriority agenda in Ethiopia, neonates’ risk of dying is unacceptable and one of the ten countries which accounts to two-third of global neonatal death. The magnitude and risk of death in the referral care facility was not well studied in the study area. This study was aimed to estimate neonatal death and its determinant.

**Methods:**

a prospective cohort study was conducted from November 2016 to January 2018 among neonates admitted to Dilla University Referral Hospital Neonatal Intensive Care Unit. We generated descriptive statistics and Cox-proportional hazard model to identify independent risk factors of neonatal death.

**Results:**

we identified 913 neonates with 6836 person-days of follow-up. Overall, 11.6% (n = 106) deaths of neonates were recorded. The estimated hazard ratios of neonatal death were higher among neonates whose mothers did not attend ANC follow up (HR=3.23), delivery assisted by TBA (HR=2.19), and maternal age ≥ 30 years at birth (HR=2.04). Urban residence [HR=0.54], family size of ≤ 3 (HR=0.47) and family size of 4 - 6 (HR=0.49), absence of abortion (HR=0.55), absence of illness during pregnancy (HR=0.47), iron folate intake (HR=0.29), birth weight ≥ 2500 grams (HR=0.43) were found to be protective factors.

**Conclusion:**

neonatal death at referral neonatal intensive care unit was relatively high. Early management of complications, improving quality of services at neonatal intensive care unit and ensuring maternal continuum of care are recommended to increase survival of neonates. Besides, maternal and neonatal health-related factors were among the independent risk factors that need to design context-based policy and interventions.

## Introduction

Newborn health has been improved significantly in the era of Millennium Development Goal even though the target of reducing neonatal mortality by two-thirds was not achieved [1], with an estimated 2.6 million deaths of newborns within 28 days of life in the world in 2016 [2]. Moreover, neonatal mortality reduction has been slower (47%) compared to the progress of under-five mortality reduction (58%) and it constitutes 45% (44.9%-45.2%) of the total under-five deaths [3-5]. By 2030, the death and share of neonatal death are expected to rise to 69 million deaths and 52 percent respectively [3, 6]. Hence, neonatal health becomes the agenda of Sustainable Development Goals (SDGs) with an entitled target of reducing neonatal mortality below 12 per 1,000 live births by 2030 [3, 6-8]. Thus, ensuring newborn survival has become the most critical intervention to the overall childhood survival.

Ethiopia had one of the world's highest neonatal mortality rate (NMR) (28.1 per 1000 live births) in 2016 [9, 10]. The NMR was higher than that of the Africa continent (27 deaths per 1,000 live births) and nine times higher than the developed countries (3 deaths per 1,000 live births), yet similar to the sub-Sahara African countries (29 deaths per 1000 live births) [3]. The NMR also varies among different socioeconomic strata and geographic regions of the country [11-14]. As a result, the federal ministry of Ethiopia designed neonatal survival strategies including establishments of the basic newborn care units (newborn corners) at primary health care centers and neonatal intensive care units (NICUs) at hospitals [15, 16]. These strategies are part of the global and national targets set in SDGs. However, the maternal and neonatal health of the country bears various health system bottlenecks [15-17] as so do other low and middle-income countries in the world [18].

To achieve SDGs, it needs to understand the multifactorial and inter-related determinants of neonatal death. Understanding the risk factors of neonatal mortality gives an important public health insight [3]. Several studies indicated maternal, neonatal, health system, and socioeconomic risk factors were related to neonatal survival. These factors could easily preventable through the provision of the continuum of care from pregnancy to delivery and to the immediate postnatal period [11-14, 19-22]. However, empirical evidence assessing the risk factors of neonatal death is lacking from the prospective cohort of neonates in Ethiopia. Available evidence generated so far were either from hospital-based secondary records or retrospective surveys with methodological limitations. Therefore, the present study aimed to estimate neonatal death (time to death) and assess the independent risk factors of neonatal death.

## Methods

**Study design, setting, and populations:** a prospective cohort study was conducted among neonates admitted to Dilla University Referral Hospital Neonatal Intensive Care Unit (NICU) from November 2016 to January 2018. Dilla University referral hospital serves as a referral hospital for all districts in Gedo zone and Abay district of Oromia region and Dara District in Sidama zone. In Gedo zone, only Dilla University Referral Hospital Neonatal Intensive Care Unit is established. All neonates admitted to the neonatal care unit from Dillazuria, Wonago, Bule, Gedeb, Kocere, Yirgachefee, Dilla town, Abay and Dara districts were followed from cohort entry up to the occurrence of an event (death) or end of follow-up.

**Recruitment and interviewing of study participants:** structured and interview-administered questionnaire adopted from previous research and WHO standard questionnaires [23] was used to prospectively collect the data. The mothers of index neonates were the respondent for the interview in addition to the data collected from the clinical charts. Ten trained data collectors interviewed with mothers of index children and gathered clinical information by reviewing their clinical charts. Admitted neonates were daily visited by the data collectors. They followed the neonates from admission to death or discharge and the alive neonate was censored at the end of the study period.

**Measurements of variables:** the data that were collected prospectively consisted of time to death of neonates (dependent variable), and the socio-demographic, economic, neonatal, and maternal and health service-related characteristics (independent variables). The outcome variable was dichotomized to dead and censored for survival analysis. Neonatal death is defined as any death occurring during the first 28 completed days of life, with early neonatal deaths being those occurring in the first 7 days and late neonatal deaths those 8 to 28 days after birth. A final assessment of admission diagnosis and causes of death were set by physicians after conducting the necessary clinical and laboratory investigation.

**Data management and analysis:** we checked the data integrity using Epi Info version 7 and analyzed using statistical package for social sciences version 20. We generated descriptive statistics using the frequency distribution for categorical variables and using mean for continues variables. We assessed factors associated with neonatal death using a univariable and multivariable Cox-proportional hazard model. All variables having p-value, < 0.05 were considered as candidates for the final model.

**Ethics approval:** the study was approved by the health research and ethics review committee of the College of Health Sciences and medicine, Dilla University. Moreover, the respondent gave voluntary written consent before the interview. The information obtained was kept anonymous and thereby assured of confidentiality.

## Results

**Characteristics of mothers and neonates:** overall, 987 neonates admitted to the NICU of Dilla University Referral Hospital. We included 913 neonates into the analysis and excluding those newborns with age greater than 28 days (n=74) at the time of admission. The majority of mothers (n=341, 37.3%) were between in the age group of 24-29 years in the last birth with a mean (±SD) age of 26.6 (±4.7) years. Three-fourths of mothers (75%) completed primary and high school education and about 58.6% of them were housewives (Table 1).

**Table 1:** socio-demographic characteristics of mothers among admitted neonates to Dilla University Referral Hospital Neonatal Intensive Care Unit, from November 2016 to January 2018

Variables		Number (%)
Age of mothers at the current pregnancy	Mean (SD)	26.6 (± 4.7)
	<24 Years	287 (31.4)
	25-29 Years	341(37.3)
	> 30	285 (31.2)
Marital status	Married	891(97.6)
	Other*	22 (2.4)
Districts of the respondents	Dillazuria	119 (13.0)
	Wonago	108 (11.8)
	Bule	81 (8.9)
	Gedeb	79 (8.7)
	Kochere	84 (9.2)
	Yiregacheffe town and district	131 (14.3)
	Dilla town	154 (16.9)
	Abay District, oromay region	82 (9.0)
	Dara District, Sidama zone	75 (8.2)
Place of residence	Urban	316 (34.6)
	Rural	597 (65.4)
Mother’s educational status	Uneducated	168 (18.4)
	Primary school (Grade 1-8)	324 (35.5)
	High school (Grade 9-12)	362 (39.5)
	Diploma and above	59 (6.5)
Husband /or partner educational status	Uneducated	75 (8.2)
	Primary school (Grade 1-8)	232 (25.4)
	High school (Grade 9-12)	325 (35.6)
	Diploma and above	281 (30.8)
Mothers’ occupational Status	Government or private employee	52 (5.7)
	Self-employed including merchant	326 (35.7)
	Housewife	535 (58.6)
Husband /or partner occupation	Government or private employee	378 (41.4)
	Self-employed including merchant	303 (33.2)
	Unemployed including daily laborer	59 (6.5)
	Farmer	173 (18.9)
Family size	Mean (SD)	4.01 (± 2.3)
	Less than three family	495 (54.5)
	4-6 family	272 (29.8)
	Greater than seven family	146 (16..0)

SD: Standard Deviation, other* includes: Single, widowed and divorced,

Even though most of the mothers (n=776, 85%) reported to have at least one antenatal care (ANC) visit during their last pregnancy, more than half of them (n=518, 56.7%) had less than four ANC visits (focused ANC). About (n=496, 54.3%) women gave their last birth at government hospitals while 327 (35.8%) women´s last delivery was at primary health care centers. Two hundred forty-eight mothers (27.2%) had experienced a complication during delivery while 241 (26.4%) of mothers had experienced a complication during pregnancy period. Bleeding (n=87, 23.8%), visual problem (n=74, 20.2%), and hypertension (n=61, 16.7%) were the most commonly reported complications during the prenatal period (Table 2). There was a male predominance of admission in the NICU (n=510, 55.9%). Most of the admissions (n=788, 86.3%) occurred during the first week of life. Above two-thirds of neonates were term babies (67.5%). The mean weight of neonates at admission and discharge was 2737.7 (SD=±839.8) and 2878.8 (SD=±1031) grams, respectively (Table 3).

**Table 2:** reproductive and obstetric characteristics of mothers among admitted neonates to Dilla University Referral Hospital Neonatal Intensive Care Unit, from November 2016 to January 2018

Variables		Number (%)
ANC visit in the last pregnancy	< 4 ANC visit	518 (56.7)
	> 4 ANC visit	258 (28.3)
	No ANC visit	137 (15)
HEW visit during pregnancy	1-2 visit	132 (14.5)
	>3 Visit	31 (3.4)
	Not visited by HEW	750 (82.1)
Iron folate taken during pregnancy	Mean (SD)	57 (±16.15)
	Less than 60 days	445 (48.7)
	60 days and above	304 (33.3)
	Not taking at all	164 (18.0)
Place of delivery	Hospital	496 (54.3)
	Health center	327 (35.8)
	Private clinic	30 (3.3)
	Home	60 (6.6)
Mode of delivery	Vaginal delivery	691 (75.7)
	Assisted delivery	70 (7.7)
	Cesarean section	152 (16.6)
Delivery assistant	Skilled personnel	848 (92.9)
	Family/or relatives and TBA	65 (7.1)
Complication of labor	Yes	248 (27.2)
	No	665 (72.8)
Complication of pregnancy	Yes	241 (26.4)
	No	672 (73.6)
Type of disease diagnosed in last pregnancy	Hypertension	61 (16.7)
	DM	16 (4.4)
	Asthma	6 (1.6)
	Anemia	35 (9.6)
	Malaria	5 (1.4)
	Headache	57(15.6)
	Visual problem	74 (20.2)
	Pregnancy-related hypertension	25 (6.8)
	Bleeding	87 (23.8)
Number of alive children	Mean (SD)	3.02 (±2.05)
	Less than two children	294 (32.2)
	Three to four children	154 (16.9)
	Greater than five children	142 (15.6)
	No children	323 (35.4)
History of abortion	Yes	74 (8.1)
	No	839 (91.9)
History of still birth	Yes	98 (10.7)
	No	815(89.3)

TBA: Traditional Birth Attendant, SD: Standard Deviation, ANC: Antenatal care, HEW: Health Extension Worker

**Table 3:** neonates characteristics among admitted neonates to Dilla University Referral Hospital Neonatal Intensive Care Unit, from November 2016 to January 2018

Variables		Number (%)
Age of the neonates at admission (days)	Mean (SD)	4.4 (± 4.03)
	1-7 days	788 (86.3)
	7-14 days	79 (8.7)
	14-21 days	40 (4.4)
	21-28 days	6 (0.7)
Sex of neonate	Male	510 (55.9)
	Female	403 (44.1)
Weight of the neonates at birth	Mean (SD)	2639.1 (+ 818.4)
	< 2500 grams	289 (31.7)
	>2500 grams	426 (46.7)
	I don’t know	198 (21.7)
Weight of the neonates at admission	Mean (SD)	2737.7 (±839.8)
	< 2500 grams	342 (37.5)
	>2500 grams	571 (62.5)
Weight of the neonates at discharge	Mean (SD)	2878.8 (±1031)
	< 2500 grams	320 (35.0)
	>2500 grams	593 (65)
Gestation age at birth	Mean (SD)	37.2 (± 2.4)
	Preterm (< 37 weeks	297 (32.5)
	Term (>37 weeks)	616 (67.5)
Duration of stay in the NICU	Mean (SD)	7.5 (± 4.07)
	Seven and less than seven days	619 (67.8)
	Greater than seven days	294 (32.2)

SD: Standard Deviation, NICU: Neonatal Intensive Care Unit

**Magnitude and timing of death among admitted neonates:** overall, 11.6% (n=106); (95% CL (9.6-13.7) of neonates died during the follow-up. The overall incidence of neonatal mortality was 15.5 per 1000 person- days. We found that 36(34.0%); 95% CL (25.5-43.4) and 65(61.3%); 95% CL (51.9-69.8) of deaths occurred during the first and second weeks of neonatal life, respectively. Late neonatal death (n=70, 66.0%); 95% CL (56.6-74.5) was higher compared to early neonatal deaths (n=36, 34.0%); 95% CL (25.5-43.4).

**Figure 1:**
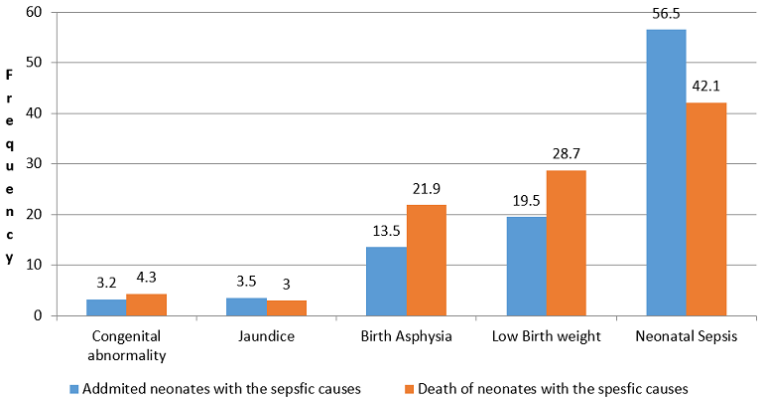
clinical diagnosis and contribution to deaths in Neonatal intensive care unit at referral Hospital in Godeo Zone, from November 2016 to January 2018; a neonate having more than one diagnosis is counted more than once

**Neonatal illness and cause-specific neonatal death during the follow-up:** the most common cause of admission was neonatal sepsis (56.5%) followed by low birth weights (19.5) while the least common was congenital malformation (3.2%). Sepsis, (n=98, 42.1%), low birth weight (LBW), (n=67, 28.8%) were the main leading cause of deaths. Birth asphyxia was not the major cause of admission, but it accounted for a higher proportion (21.9%) of neonatal death (Figure 1).

**Risk factors for neonatal death:** both binary Cox-regression and multivariable Cox-regression analysis were carried out to identify risk factors of neonatal mortality (Table 4). In the multivariable Cox-regression model; maternal age greater than 30 years (HR=2.04, 95% CI: 1.13-3.66), delivery assisted with TBA and/or family (HR=2.19, 95% CI: 1.18-4.10) and neonates whose mothers did not have ANC follow up visit (HR=3.23, 95% CI: 1.06 -9.84) carried a high risk of neonatal death. However, mothers who lived in urban area (HR=0.54, 95% CI: 0.27-0.93), household family size less than three (HR=0.47, 95% CI:0.22-0.98) and 4 up to 6 family (HR=0.49, 95% CI: 0.26-0.94), no history of abortion (HR=0.55, 95% CI: 0.30-0.92), mothers who did not have maternal illness during prenatal period (HR=0.47, 95% CI: 0.30-0.74), mothers who took Iron folate tablets (HR=0.29, 95% CI: 0.09-0.89) and birth weight >2500 grams (HR=0.43, 95% CI: 0.24-0.77) remained as a lower risk for neonatal death among admitted neonates (Table 4).

**Table 4:** adjusted hazard ratios of newborn related risk factors associated with neonatal death among admitted neonates to Dilla University Referral Hospital Neonatal Intensive Care Unit, from November 2016 to January 2018

Variables		Neonatal death		Unadjusted HR (95% CI)		Adjusted HR (95% CI)
**Yes, n=807**	**No, n=106**
Place of residence	Urban	297(36.8)		**0.42**	**0.25-0.68**	**0.54 (0.27-0.93)**
	Rural	510 (63.2)		1		1
Mothers educational status	Uneducated	137(17.0)		1		1
	Primary school (1-8 grade)	280 (34.7)	44 (41.5)	0.68	0.43-1.08	0.67 (0.26-1.71)
	High school and above	391(48.5)	30 (28.3)	**0.35**	**0.22-0.58**	0.77 (0.35-1.69)
Husband educational status	Uneducated	62 (7.7)	13 (12.3)	1		**1**
	Primary school (Grade 1-8)	188 (23.3)	44 (41.5)	1.05	0.57-1.96	1.3 (0.59-2.85)
	High school (Grade 9-12)	297 (36.8)	28 (26.4)	**0.45**	**0.23-0.86**	0.64 (0.25-1.62)
	Diploma and above	260 (32.2)	21 (19.8)	**0.37**	**0.18-0.73**	0.46 (0.16-1.49)
House hold family number	Less than three family	454 (56.3)	41 (38.7)	**0.38**	**0.24-0.62**	**0.47 (0.22-0.98)**
	4-6 family	236 (29.2)	36 (34.0)	**0.57**	**0.35-0.93**	**0.49 (0.26-0.94)**
	Greater than seven family	117 (14.5)	29 (27.3)	1		1
Age of mothers at last birth	<24 Years	271 (33.6)	16 (15.1)	1		**1**
	25-29 Years	293 (36.3)	48 (45.3)	**2.33**	**1.33-4.14**	0.82 (0.38-1.76)
	>30 years	243 (30.1)	42 (39.6)	**2.56**	**1.44-4.56**	**2.04 (1.13-3.66)**
Gestation age at birth	Preterm (< 37 weeks	251(31.1)	46 (43.4)	**1.81**	**1.24-2.67**	1.11 (0.67-1.85)
	Term (>37 weeks)	556 (68.9)	60 (56.6)	1	1	1
History of abortion	Yes	82 (10.2)	16 (15.1)	1		**1**
	No	725 (89.8)	90 (84.9)	**0.41**	**0.24-0.68**	**0.55 (0.30-0.92)**
Who Assist during Delivery	TBA and family/or relatives	51(6.3)	16 (13.2)	**2.16**	**1.23-3.78**	**2.19 (1.18-4.10)**
	Skilled personnel	756 (93.7)	92 (86.8)	1		1
Maternal illness during pregnancy	Yes	192 (23.8)	49 (46.2)	1		**1**
	No	615 (76.2)	57 (53.8)	**0.35**	**0.24-0.52**	**0.47 (0.30-0.74)**
ANC follow up	Yes	695 (86.1)	81 (76.4)	1		**1**
	No	112 (13.9)	25 (23.6)	**1.89**	**1.20-2.96**	**3.23 (1.06-9.84)**
Iron folate taken	Yes	667 (82.7)	82 (77.6)	**0.43**	**0.24-0.76**	**0.29 (0.09-0.89)**
	Not taking	140 (17.3)	24 (22.6)	1		1
Weight of neonates at birth	< 2500 grams	245 (30.4)	44 (41.5)	1		**1**
	>2500 grams	397 (49.2)	29 (27.4)	**0.41**	**0.26-0.66**	**0.43 (0.24-0.77)**
	I don’t know	165 (20.4)	33 (31.1)	0.83	0.53-1.30	0.95 (0.52-1.75)

ANC: Antenatal care, HEW: Health Extension Worker,

## Discussion

In this current study, we found that nearly one in every ten (11.6%) (95% CI: 9.6 - 13.7) infants were dying before celebrating their first month of life (neonatal period). This findings was higher compared to a study done in Tigray region-Ethiopia, (6.25%) [24], but lower compared to the overall estimated national neonatal mortality rate (29 per 1000 live births) for Ethiopia [10]. The higher death in this study might be neonates transferred to Neonatal ICU for admission might have risk of death.

In this study, shorter survival time (most of the neonatal death) found in the earlier age of neonatal life, which was in line with the previous studies [12, 24, 25]. This might be because preterm or severely sick newborn die early. In the study, we found that LBW contributing to being most of the neonatal deaths, (28.8%), this could be an alarm to health professionals to improve quality care at the time of birth and special care for sick and small newborns. Previous evidence also reported that poor or low availability quality of child health care facilities could further worsen the risk of death [26, 27].

Neonatal sepsis and low birth weights were the leading causes of neonatal death and hospital admission, which was consistent where the studies did Ethiopia and abroad [1, 10, 28, 29]. In the study, nearly one-third of deaths (33.1%) were recorded with the cause of sepsis and also 44.7% of deaths were attributed to by LBW. Therefore, this study alert for the establishment of advanced NICU and strengthens the referral systems. Besides, antenatal and intrapartum monitoring of high-risk pregnancy, care during labor and delivery as well as immediate neonatal care practices including adequate basic neonatal resuscitation care at the time of birth should be the highest priority intervention in the study area.

An infant born in an urban area has a lower risk of death than an infant born in a rural area. The reason may be those mothers who lived in the urban area have an awareness of the advantage of maternal health care service utilization to mothers and their babies during pregnancy, labor, immediate and early neonatal periods and that has an effect on child survival in the postnatal period. Maternal and neonatal health conditions have different impacts on neonatal survival status. The study showed that neonates whose mothers did not have the history of abortion; did not experience maternal illness during the prenatal period, and neonates whose mother took Iron foliate and neonates whose birth weight >2500 grams have a lower risk for neonatal death. Similar to previous studies done in Ethiopia [24, 30], neonates whose mothers completed high school and above had a lower risk of death compared to uneducated mothers. However, after adjusting for other variables, the risk of death didn't vary significantly by educational status. In the study, neonates born to a mother who was greater than 30 years of age have a higher risk of death, like other studies conducted in Ethiopia [12].

On the other hand, variables such as infrequent or lack of ANC visit and delivery assisted with TBA and family members have a higher risk of neonatal death. Several previous studies [30, 31, 32] have also reported that lack of adequate and quality ANC visit that results in the inadequate monitoring of pregnancy, maternal and neonatal complication during and after delivery, which was associated with increased the risk of neonatal death. Moreover, maternal healthcare service is an important predictor of neonatal survival. Therefore, neonatal outcome depends on maternal health during the pregnancy period. Therefore, this finding suggests that special consideration needs to be given for maternal obstetric care during pregnancy.

## Conclusion

The present study reported that the magnitude of neonatal death was (11.6%); (95% CI: 9.6 - 13.7), which is one of the highest and nearly threefold of the national estimated neonatal mortality rate, which seek more attention in order to meet the goal of child survival in Ethiopia to 11 per 1000 live births by 2020. Most of the neonatal death found in the earlier age of neonatal life. The risk of dying among admitted neonates is determined by both maternal socio-demographic and obstetric characteristics, and neonatal birth conditions. The leading causes of deaths, admission, and predictors of death were related to maternal socio-demographic and obstetric characteristics that could be improved a continuum of care to women during pregnancy, labor, immediate and early neonatal periods. The neonatal intensive care unit should work on early diagnosis, appropriate management and continuous care. Therefore, a responsive health care system that is equipped with lifesaving commodities and well-trained staff is the priority intervention in our setup.

### What is known about this topic

- We found that nearly one in every ten infants was dying before celebrating their first month of life (neonatal period);
- Most of the neonatal death found in the earlier age of neonatal life.

### What this study adds

- The risk of dying among admitted neonates is determined by both maternal socio-demographic and obstetric characteristics, and neonatal birth conditions;
- A responsive health care system that is equipped with lifesaving commodities and well-trained staff is the priority intervention in our setup.
